# Synergistic Effects of Localized Surface Plasmon Resonance, Surface Plasmon Polariton, and Waveguide Plasmonic Resonance on the Same Material: A Promising Hypothesis to Enhance Organic Solar Cell Efficiency

**DOI:** 10.3390/nano13152209

**Published:** 2023-07-29

**Authors:** Issoufou Ibrahim Zamkoye, Bruno Lucas, Sylvain Vedraine

**Affiliations:** University of Limoges, Centre National de la Recherche Scientifique, XLIM, UMR 7252, F-87000 Limoges, France; bruno.lucas@unilim.fr

**Keywords:** plasmonic resonance, LSPR, SPP, silver nanowires, organic solar cells, finite-difference time-domain simulation, electrode

## Abstract

This work explores the utilization of plasmonic resonance (PR) in silver nanowires to enhance the performance of organic solar cells. We investigate the simultaneous effect of localized surface plasmon resonance (LSPR), surface plasmon polariton (SPP), and waveguide plasmonic mode on silver nanowires, which have not been thoroughly explored before. By employing finite-difference time-domain (FDTD) simulations, we analyze the plasmonic resonance behavior of a ZnO/Silver nanowires/ZnO (ZAZ) electrode structure. Our investigations demonstrate the dominance of LSPR, leading to intense electric fields inside the nanowire and their propagation into the surrounding medium. Additionally, we observe the synergistic effects of SPP and waveguide plasmonic mode, contributing to enhanced light absorption within the active layer of the organic solar cell. This leads to an improvement in photovoltaic performance, as demonstrated by our previous work, showing an approximate 20% increase in photocurrent and overall power conversion efficiency of the organic solar cell. The incorporation of metallic nanostructures exhibiting these multiple plasmonic modes opens up new opportunities for improving light absorption and overall device efficiency. Our study highlights the potential of these combined plasmonic effects for the design and optimization of organic solar cells.

## 1. Introduction

The interaction of light with certain nanoscale conductive materials can induce a well-known phenomenon in the literature, referred to as plasmonic resonance (PR). At a specific wavelength for each material with enough available electrons, an electric field is created due to the oscillation of conduction electrons around the atoms of the material. These electron oscillations are called surface plasmons, and the nanomaterials that support surface plasmons are referred to as plasmonic materials [1,2,3]. This phenomenon is widely recognized in the fields of physics, chemistry, and materials science due to its diverse range of potential applications, including optical sensing, light generation, solar cells, biomedicine, and electronics. With its growing popularity, SPR has become a significant focus of research aimed at understanding its properties and exploring its potential applications. The discovery of the SPR phenomenon dates back over a century to Wood [4]. Wood observed anomalous reflected structures when polarized light was projected onto metalized diffraction grating [4]. Lord Rayleigh [5] attempted to elucidate the phenomenon a few years later (in 1907). The physical interpretation was not resolved until 1968 by Otto [6]. Also in 1968, Kretschmann and Raether [7] described the excitation of surface plasmons (SP) using a specific optical setup. The specificity of this excitation is the presence of a plasmon at the interface of the material with its surrounding media, not only inside the material.

In organic photovoltaic (OPV) devices, the relatively low mobility of charge carriers (around 10^−4^ cm^2^·V^−1^·s^−1^) and the short diffusion length in most organic semiconductor materials [8] necessitate the use of thin active layers, typically around 100 nm or less, to enhance charge diffusion and extraction [9]. However, this thin active layer leads to poor absorption of incident light, resulting in the significant energy loss of photons that could otherwise be converted into electrical energy. Extensive research has therefore been conducted to improve light absorption in OPVs. Multiple studies have demonstrated that surface plasmon resonance (SPR) can be introduced in OPVs through the incorporation of metallic nanostructures, without varying the thickness of the active layer. The introduction of SPR amplifies the light collection in the active layer, thereby enhancing photovoltaic performance [1,10,11,12,13,14]. In fact, the absorption of the active layer is directly proportional to the square of the electric field, as described by the following equation [15]:

(1) $$Q\left(z,\lambda \right)=\alpha \left(\lambda \right)\frac{n_{i}}{n_{0}}\frac{{\left|E\left(z\right)\right|}^{2}}{{\left|E_{0}\right|}^{2}}$$

Here, *Q*(*z*, *λ*) represents the absorption as a function of the position (*z*) within the device stack, wavelength (*λ*), *α*(*λ*) denotes the absorption coefficient, *n_i_* and *n*_0_ are the refractive indexes of the *i* layer and the surrounding medium, and *E*(*z*) and *E*_0_ represent the electric fields at position *z* and the incident electric field, respectively.

This equation establishes a direct relationship between the absorption of the active layer and the electric field resulting from the surface plasmon resonance. However, it should be noted that the propagation of this electric field is limited in terms of distance, as further discussed in this investigation. By enhancing absorption, the generation of electrons can be improved, consequently enhancing the external quantum efficiency. Alternatively, achieving similar absorption with a thinner active layer can mitigate the challenges associated with charge diffusion length commonly observed in organic solar cells. Furthermore, this effect can be exploited in organic solar cells with a thick active layer for better efficiency [1].

### 1.1. Far-Field Plasmonic Effects: Scattering

Far-field scattering results in the optical path of incident light being lengthened, which can enhance photon absorption. The size of the metal nanoparticles that best matches the far-field scattering effect is generally greater than 30 nm [16].

For plasmonic nanostructures embedded in layers at the interface of transparent electrodes (i.e., HTL (hole transport layer) in conventional-type solar cells or ETL (electron transport layer) in inverted-type cells), forward scattering is required. The geometry and interface of plasmonic nanostructures can be optimized to minimize surface reflection. In addition, the optical paths of incident sunlight in the absorbing medium and their interaction time are thus increased, resulting in improved absorption efficiency of the active layer [17].

The direction of scattering can be adjusted by controlling the size and geometry of the plasmonic nanostructures. Starting with nanoparticles around 50 nm in diameter, photons are scattered forwards and backwards in comparable quantities [18]. As particle size increases, more radiation is scattered backwards.

Nanoparticles (NPs) whose size is below the above-mentioned intermediate range exhibit low scattering, which induces insufficient optical absorption in the active layer linked to this component. In addition, the conductivity of the device is negatively affected as more charge-trapping sites are formed at the interfaces of the NPs due to their large surface-to-volume ratio. For plasmonic nanostructures integrated outside the active layer, these losses cancel out the possible advantages of small NPs (e.g., ~20 nm), which can accentuate the coupling of the plasmonic field close to the active layer [19].

### 1.2. Near-Field Plasmonic Effects

**Figure 1:**
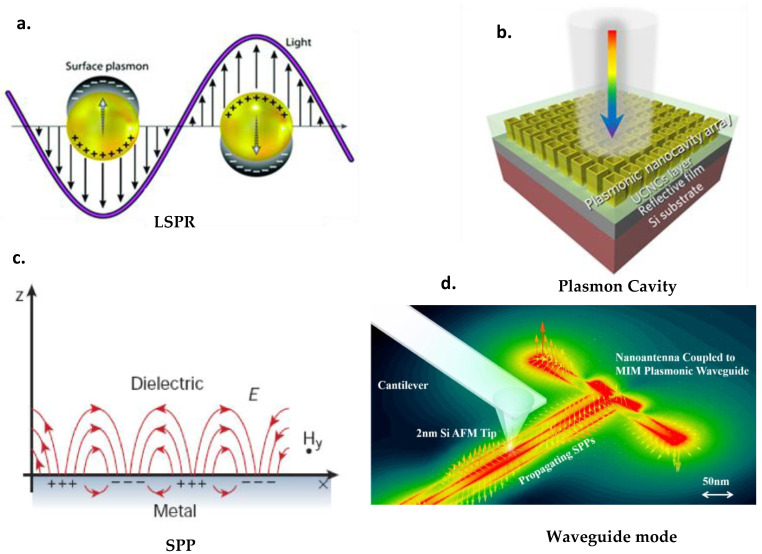
(**a**) representation of the surface plasmon localized on the nanoparticles [20], (**b**) plasmon cavity [21]; (**c**) schematic diagrams illustrating a surface plasmon polariton (SPP) [22]; (**d**) plasmon waveguide [23].

There are four modes of plasmonic resonance (Figure 1), which can be classified into two groups: propagative and non-propagative modes.

#### 1.2.1. Non-Propagating Modes

**Localized Surface Plasmon Resonance (LSPR)**

When a surface plasmon is confined to a NP of a size smaller than the wavelength of the incident light, the free electrons of the NP participate in the collective oscillation, which is called localized surface plasmon resonance (LSPR, Figure 1a). The LSP has two important effects. Firstly, the electric fields near the particle surface are strongly strengthened, this strengthening being greatest at the surface and decreasing rapidly with distance [20]. Secondly, the particle’s optical radiation achieves maximum capacity at the plasmon’s resonance frequency. The resonance frequency depends on the geometry of the metal nanostructures. Consequently, appropriate adaptation of nanoparticle size and morphology is an effective approach to controlling the resulting plasmon electric field radiation to meet specific demands. For noble metal nanoparticles, resonance typically occurs in the visible and near-infrared wavelength region [24,25]. LSPR often occurs when the surfaces of plasmonic nanostructures are textured [26].

NPs smaller than 30 nm act as sub-wavelength antennas during LSPR excitation. In such cases, the absorption enhancement comes mainly from the LSPR effect, rather than the scattering effect [27]. The near-field plasmon induced in the active layer increases the absorption cross-section, and thus, effectively enhances photon absorption.

**Plasmonic cavity mode**

When specific metal–dielectric configurations are built, the spectral wavelength ranges for absorption enhancement can be extended further due to plasmonic coupling modes. For example, a metal–dielectric–metal (MDM) structure is obtained when the active layer is sandwiched between the metal electrode and the metal NP layer. By appropriately selecting NP geometries, surface plasmon stationary waves can be generated due to constructive interference in the finite cavity, reducing light leakage from surface plasmon generation in the lateral gaps between NPs. Thus, the plasmon cavity mode is excited in the MDM and a strong electromagnetic field is confined to the light-absorbing layer. Interestingly, the field enhancement from the plasmon cavity mode (Figure 1b) is independent of polarization or incident light angles [28].

#### 1.2.2. Propagating Modes

**Surface plasmon polariton (SPP)**

Unlike LSPR, surface plasmon polariton (SPP, also known as SPR [29]) often occurs on or near a planar metal (Figure 1c). The SPP is an electromagnetic wave at infrared or visible frequencies that propagates along the metal–dielectric interface [30,31,32]. The charge movements of the SPP involve contributions from both the metal and the dielectric. Perpendicular to the interface, the spatial confinement of SPPs lies in a sub-wavelength scale range, resulting in a more intense electromagnetic field. The amplitude of SPP waves decreases exponentially with increasing distance in each medium from the interface. A SPP can propagate along the interface for 10–100 µm until its energy is consumed by absorption or scattering [33,34]. The increase in optical path length with lateral propagation significantly enhances absorption.

**The photonic waveguide mode**

In addition to the SPP mode, a further mode type can be excited at planar metal–dielectric interfaces during incident radiation, which also contributes to absorption enhancement. It is called the “waveguide mode” because of its similarity to the propagation model in dielectric waveguides. Along its lateral propagation, part of the waveguide mode can be absorbed by organic semiconductors, in which excitons are generated [35,36].

Excitation of the waveguide mode is independent of the polarization of the incident irradiation. This means that waveguide modes can be generated irrespective of whether the polarization direction of the excitation is parallel or orthogonal to the directions of the metal nanostructures [37].

Given the architecture of the device and the structure of the metal nanostructures to be used, some modes will be much more favorable than others. In our case, we are using metal nanowires that have a cylindrical shape with a diameter of 35 nm and a length of 25 µm based on our previous investigation showing an experimental increase in the efficiency of an organic solar cell despite its thick active layer [1]. Later in this article, we will look at the modes that are predominant in this type of structure.

In this work, we will first explore the concept of plasmonic resonance, followed by the study of plasmonic resonance in silver nanowires using finite-difference time-domain (FDTD) simulation software, in which a ZnO/AgNWs/ZnO (ZAZ) electrode model will be constructed with the different materials used. We will observe the spatial distribution of the electric field as a function of wavelength and the type of plasmonic resonance.

## 2. Methods

**Figure 2:**
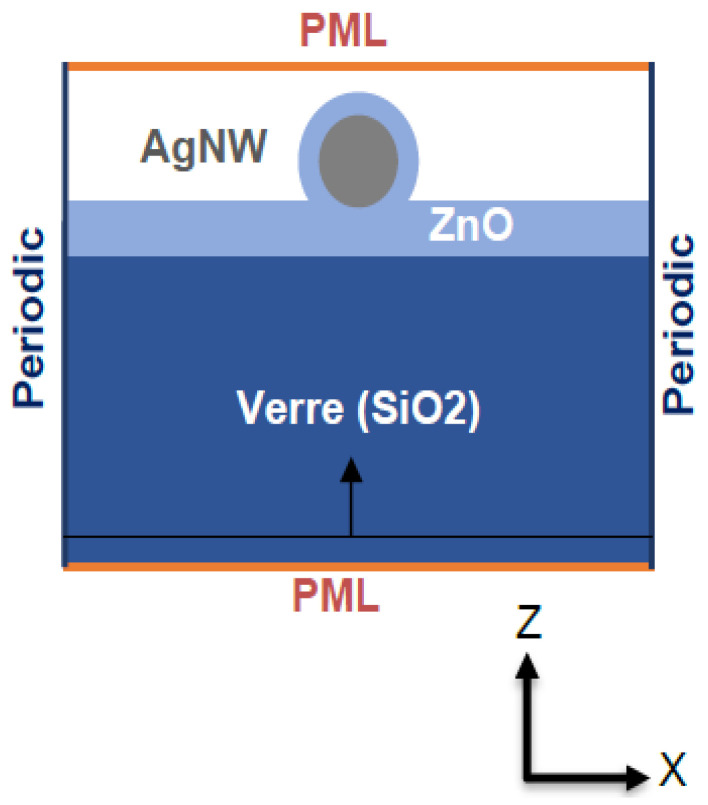
Structure of the numerical model of the ZAZ electrode and boundary conditions.

The ZAZ electrode was modeled according to the structure described in Figure 2. We modelized a nanowire made with silver and surrounded by an interface layer of a solar cells made with ZnO. We modeled the substrate with silica glass (SiO_2_). The optical indexes used to model ZnO and silver nanowires (AgNWs) were taken from references [38,39], respectively. SiO_2_ is characterized by an almost constant real optical index equal to 1.46 and 0 imaginary part in the visible spectrum [11]. All of the materials used in this configuration have been chosen to ensure the photons pass through to reach the active layer. As a result, they are all transparent to visible light except for the AgNWs, which individually reflect visible light; however, when deposited to form a ZAZ electrode they exhibit high transmission [1].

All of our simulations are performed in three dimensions. The simulation area is delimited by PML (perfect matched layer) boundary conditions along the *z*-axis, periodic along the *x*-axis, and Bloch along the *y*-axis. The spatial mesh size is 0.25 nm, minimum, and the time mesh size is 3.36 × 10^−18^ s. The surrounding medium is air, and the illumination source consists of two polychromatic plane waves polarized at 90° to each other, with the aim being to obtain unpolarized illumination. Various monitors were introduced into the simulation to observe the transmission and spatial distribution of the electric field around the AgNWs.

## 3. Result and Discussion

### 3.1. ZAZ Electrode Modeling with One Silver Nanowire

**Figure 3:**
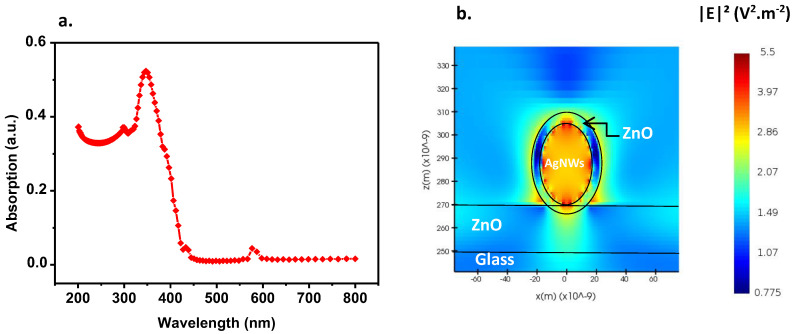
(**a**) absorption of the modeled ZAZ electrode; (**b**) spatial distribution of the squared modulus of the electric field around AgNW at peak absorption wavelength (347 nm).

In this section, we investigate plasmon resonance around a single silver nanowire in order to observe the behavior of the electric field as a function of the wavelength. The diameter of the silver nanowire is 35 nm, which corresponds approximately to the diameter of the AgNWs used experimentally [1]. The lower layer of ZnO nanoparticles (20 nm) is located below the AgNW and the upper layer is modeled by the ring around the AgNW with a diameter greater than 45 nm (Figure 3a).

Figure 3a shows the absorption of the ZAZ electrode, with an absorption peak at approximately 347 nm (typical resonance of silver nanosphere). Figure S3 illustrates the transmission of the ZAZ electrode in comparison to the experimental ZAZ, revealing a close similarity in transmission characteristics between the numerical and experimental ZAZ. The resulting electric field is shown in Figure 3b. The localized field inside the AgNW is intense due to the absorption of the nanowire at the chosen wavelength. A high electric field intensity is noted at the top of the AgNW/ZnO interface, given the difference in permittivity between the two materials, but a low intensity on the edges. Its radiation extends approximately 30 nm from the ZnO interface and spreads out into the air. The ZnO layer seems to favor plasmon propagation in air, as the electric field is stronger there than in ZnO. This effect is particularly interesting and will enable us to take advantage of this surface plasmon in the active layer that will fill this space.

Regarding the small absorption peak observed near 575 nm, it is important to note that this peak is a result of a numerical artifact related to the fit of the refractive index of the silver used in the electrode. It does not significantly affect the overall performance or application of the electrode. The main absorption peak of ZAZ, which is located at approximately 350 nm, is the primary focus for extending the absorption spectrum and enhancing the performance of organic solar cells, as mentioned in our previous work [1].

**Figure 4:**
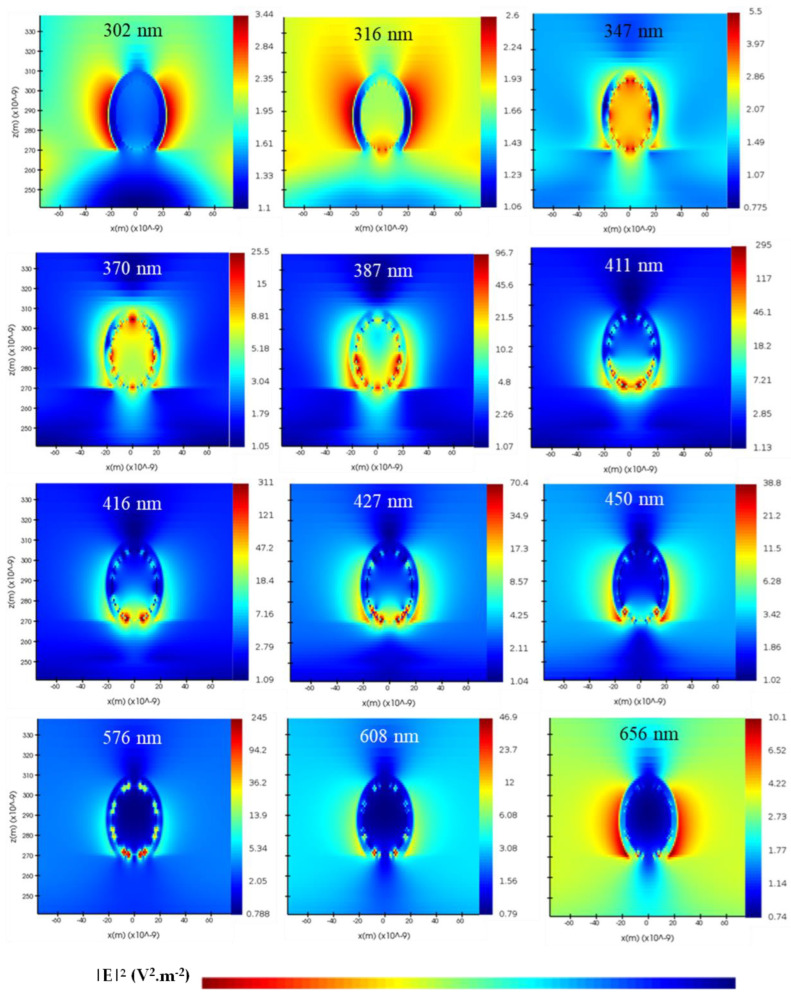
Spatial distribution of the normalized squared electric field on a section of silver nanowire at different wavelengths. We selected wavelengths that exhibit significant variations in the electric field distribution, providing a more informative representation in a concise manner.

The LSPR is the privileged plasmonic resonance mode in this configuration. A multi-wavelengths investigation (Figure 4) represents the electric field distributed around the cross-section of the silver nanowire. At 302 nm, the electric field takes the form of two lobes positioned on either side of the silver nanowire directed towards the air, under the influence of the substrate. These lobes show a strong presence of scattering compared with plasmon resonance. This radiation is very similar to that observed with a metal sphere, as demonstrated in several research works [40]. Although this electric field is localized around the AgNW, its amplitude is very low at this wavelength. At 316 nm, the electric field reaches greater distances vertically, but its intensity is still very low. Part of the electric field starts to be absorbed by the AgNW.

At 347 nm, corresponding to the AgNW absorption peak, a significant portion of the electric field remains confined within the AgNW, with a high concentration at the AgNW/ZnO interface, as mentioned in the paragraph above. Absorption inside the nanowires is limited after 387 nm, which makes it possible for most of the light to be absorbed. Radiation is almost uniform around the AgNW, with a low intensity but higher than those observed before the absorption wavelength. There is a strong localization of the field, which means a high proportion of plasmonic resonance.

Beyond 347 nm, we see a significant increase in the electric field up to 608 nm from the majority of lobes due to scattering. At 416 nm, we find the maximum electric field intensity (311 V^2^·m^−2^). The electric field distribution changes shape as a function of wavelength. This is due to the interaction of light with silver being strongly related to wavelength. For wavelengths between 347 and 387 nm, the field distribution is almost uniform around the AgNWs. However, for wavelengths above 387 nm, we note an increasingly localized distribution towards the lower section of the AgNW. High-intensity hotspots at the AgNW/ZnO interface reflect very intense local confinement of the electric field at certain wavelengths.

All of the electric field exaltation phenomena contribute to increasing the absorption of the active layer of an organic solar cell over a wide wavelength spectrum, as long as the field is present beyond the ZnO.

We have observed the behavior of the electric field on the cross-section of a AgNW and the associated plasmonic resonance mode. We will now investigate the distribution of the electric field over the longitudinal section of the AgNW.

**Figure 5:**
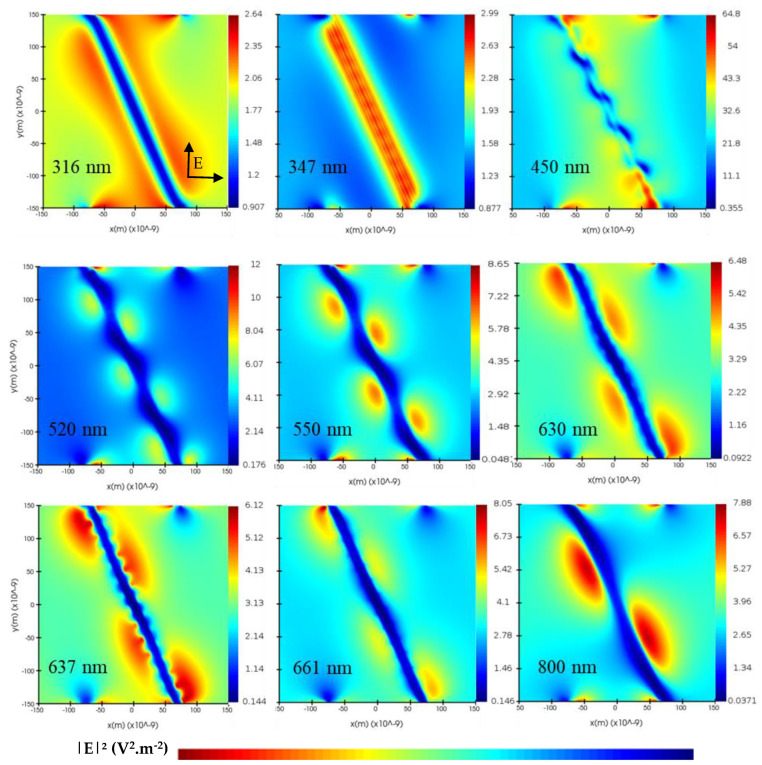
Longitudinal distribution of the normalized squared electric field 10 nm above the silver nanowire at different wavelengths. The polarization of the two plane wave sources is shown in the bottom left of the first figure. It provides a better understanding of the “diagonal” resultant representing the direction of the observed exaltations.

Figure 5 shows the longitudinal distribution of the electric field. Below the absorption wavelength, the electric field intensity is very low, as we noticed in the LSPR mode. At the absorption peak (347 nm), the electric field is confined solely to the AgNW and its surroundings.

In the following section, we develop a hypothesis to explain the interpretation of the electric field distribution along the silver nanowire.

At 450 nm, we observe a wave propagating along the AgNW with high intensity. We assume that this is the waveguide mode. This mode appears at the metal–dielectric interface (Figure 1d) and is noted in our structures with cylindrical geometry. This also explains why we observe a strong confinement of the electric field in the lower section of the AgNWs when we observe its cross-section (Figure 4 and Figure 7f). At 520 nm, this mode continues to propagate with a different frequency and a weaker, but uniformly distributed, intensity. We obtain the same behavior for the wavelength at 550 nm.

From 630 nm onwards, a second oscillation superposed to the first oscillation of the electric field appears around the AgNW, with smaller wavelengths superposed on the waveguide mode. This is a SPP mode (Figure 5), as the waveform (visible at 637 nm) corresponds to the propagation of the SPP mode, as illustrated in Figure 1c. Furthermore, the AgNWs are coated and in contact with the ZnO, which can be likened to the Otto configuration, as discussed by the Liqun Sun group [41], enabling the attenuated total reflection (ATR) condition to optically excite the SPP mode in very restricted positions. Since the propagation and localized modes (waveguide, LSPR, and SPP) are superposed, it is difficult to distinguish the single contribution of each mode. However, we noted a positive impact of these modes, as areas of low electric field intensity, when the waveguide mode is propagating alone, present a high intensity when the SPP mode is present.

The waveguide mode continues to propagate up to 800 nm, with increasing wavelengths as it moves away from the absorption peak.

**Figure 6:**
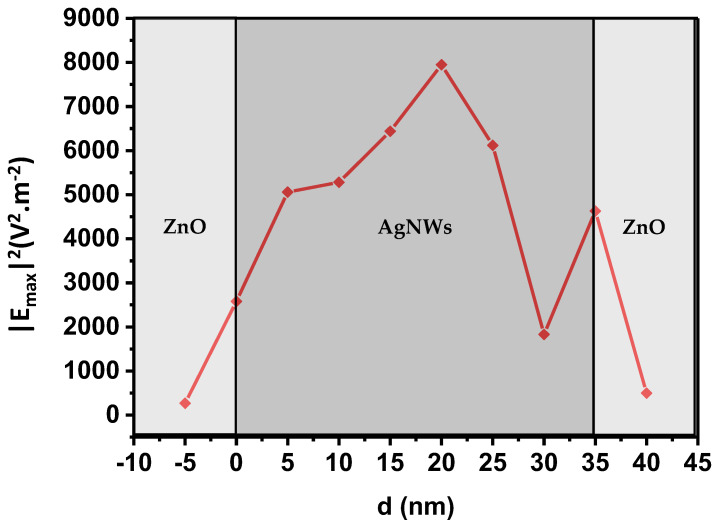
Variation of the electric field squared as a function of distance d from the observation point to the AgNW/ZnO interface (d = 0). The observation point is located at the center of the AgNWs, by vertical translation, we can move it up or down, thus varying the distance d. The monitoring wavelength is between 400 and 470 nm, depending on the location of the observation point.

Figure 6 shows the behavior of the electric field as a function of the distance between the AgNW/ZnO interface and the monitor. By varying the position of the monitor up to 40 nm, we noticed a certain increase in the electric field, which reaches its maximum value at approximately 20 nm above the interface. This seems to be in line with our hypothesis concerning the propagation of SPP and waveguide modes, since, in accordance with the Otto configuration, the distance between the dielectric and the metal is a few tens of nanometers; in our case, this distance is 20 nm.

In summary, we have demonstrated that for a silver nanowire, several plasmonic resonance modes can take place. Each of these modes can have its own peak in electric field intensity, with the LSPR mode peaking at 416 nm and the waveguide mode at 450 nm.

### 3.2. Modeling a ZAZ Electrode with Multiple Silver Nanowires

The aim of this numerical study is to propose a numerical model of the ZAZ electrode that approximates that of the experimental one, in order to demonstrate plasmon resonance. To this end, we have used a script to generate a semi-random array of ZnO-coated silver nanowires.

The numerical model structure closest to the filling factor of an experimental ZAZ electrode is that with five AgNWs. The electric field is at its maximum at 454 nm, close to the peak of the waveguide mode.

**Figure 7:**
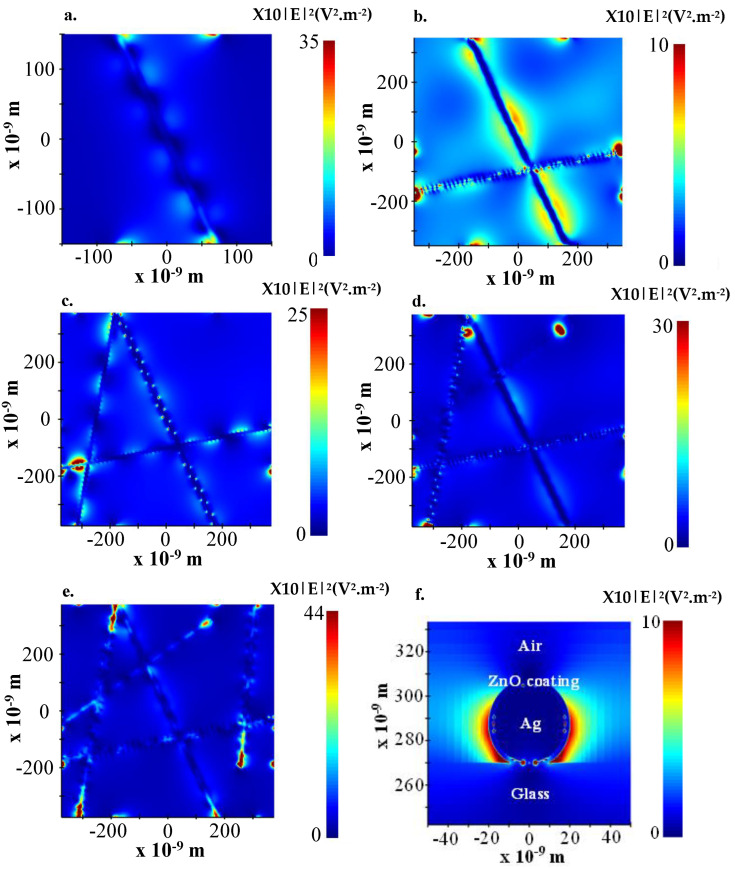
Normalized squared electric field distribution for a wavelength of 454 nm and a ZAZ electrode composed of: (**a**) one AgNW; (**b**) two AgNWs; (**c**) three AgNWs; (**d**) four AgNWs; (**e**) five AgNWs.; (**f**) normalized squared electric field distribution on the cross-section of a AgNW. The wavelength at which the field is observed is 454 nm, with the monitor at 10 nm above the AgNWs.

The two modes of plasmon propagation (SPP and waveguide) are also present for several AgNWs through the undulation of the electric field along the wires. For example, in Figure 7b we show both propagation modes, with the slightly vertical nanowire showing waveguide mode propagation and the slightly horizontal nanowire showing the superposition of both propagation modes. As we saw before, the waveguide mode is independent of polarization and the angle of incidence, which is one of the reasons why we can see it on the AgNWs, regardless of wavelength.

In Figure 7c–e, the electric field propagation around the silver nanowires differs at each AgNW/AgNW intersection. At some intersections, we see areas of low electric intensity, while at others, we see areas of very high intensity. This is due to the interference of electrical waves from plasmon resonance. At intersections with low electric field intensity, interference with waves from different AgNWs is destructive, whereas at intersections with high electric field intensity, interference is constructive.

Figure 7f illustrates the electric field distribution associated with the LSPR mode. A high electric field intensity is located on either side of the AgNW, as discussed above. This mode is clearly perceptible at the end of the AgNWs, where a strong confinement of the electric field is observed (Figure 7d,e).

The electric field transmission mode difference between horizontally and vertically placed AgNWs on the longitudinal cross-section arises from the interplay of two key factors: the polarization of the incident light wave and the interference of electric fields originating from different AgNWs. The orientation of the AgNW relative to the polarization direction of the incident light wave and the constructive or destructive interference between electric fields at the intersections of multiple AgNWs contributes to the observed variations in the electric field distribution.

The simultaneous existence of these different modes of plasmon propagation is very interesting for organic solar cells, as it would allow us to increase their absorption without increasing the thickness of the active layer.

## 4. Conclusions

In this work, we discussed the concept of plasmon resonance. There are four main modes: LSPR, SPP, waveguide, and photonic cavity. These modes can be classified as propagative and non-propagative. SPP and waveguide are classified as propagative modes, while LSPR and photonic cavity are classified as non-propagative modes. Considering the ZAZ electrode configuration, the photonic cavity mode cannot be excited due to the geometry of the AgNWs used.

To observe the electric field distribution resulting from plasmon resonance, we used the FDTD method. This enabled us to visualize the spatial distribution of the electric field across and along the cross-section of a AgNW. Excitation of the LSPR mode is evident, with significant confinement of the electric field around the cross-section of the AgNW and mainly located outside the ZnO layer. The latter functions as the role of a spacer. The distribution of the electric field along the AgNWs revealed the presence of different waves at certain wavelengths. We hypothesized that a superposition of the SPP and waveguide modes is the cause of the electric field fluctuations observed at certain wavelengths.

All of these mechanisms related to plasmon resonance are of major benefit for integration into organic solar cells to enhance their photovoltaic performance.

## Data Availability

The data that support the findings of this study are available from the 3 corresponding author upon reasonable request.

## Supplementary Materials

The following supporting information can be downloaded at: https://www.mdpi.com/article/10.3390/nano13152209/s1, Figure S1: a. configuration of the solar cell with the different interface layers, b. energy level of the materials used in the device [42], c. absorption curves for the active layer (210 nm) on ZAZ and ITO. Figure S2: J(V) curves for PF2:PC71BM (500 nm) solar cells a. under solar simulator (100 mW·cm^−2^); b. in dark conditions. Figure S3: a. Absorption and transmission curves of a ZAZ electrode modeled with 5 AgNWs; b. transmission curve of an experimental ZAZ electrode.
