# Supplementary material for: Synergistic Effects of Localized Surface Plasmon Resonance, Surface Plasmon Polariton, and Waveguide Plasmonic Resonance on the Same Material: A Promising Hypothesis to Enhance Organic Solar Cell Efficiency
Source: Nanomaterials (Basel). 2023 Jul 29;13(15):2209. doi: 10.3390/nano13152209 (PMC10421476; doi:10.3390/nano13152209)
Supplement: Supplementary file 1 [file nanomaterials-13-02209-s001.zip › nanomaterials-2500682-supplementary.pdf]

Supplementary Information:

Article

Synergistic Effects of Localized Surface Plasmon Resonance, Surface Plasmon Polariton, and Waveguide Plasmonic Resonance on the Same Material: A Promising Hypothesis to Enhance Organic Solar Cell Efficiency

Issoufou Ibrahim Zamkoye *, Bruno Lucas and Sylvain Vedraine *

University of Limoges, Centre National de la Recherche Scientifique, XLIM, UMR 7252, F-87000 Limoges, France; bruno.lucas@unilim.fr

* Correspondence: issoufou.ibrahim@etu.unilim.fr (I.I.Z.); sylvain.vedraine@unilim.fr (S.V.)

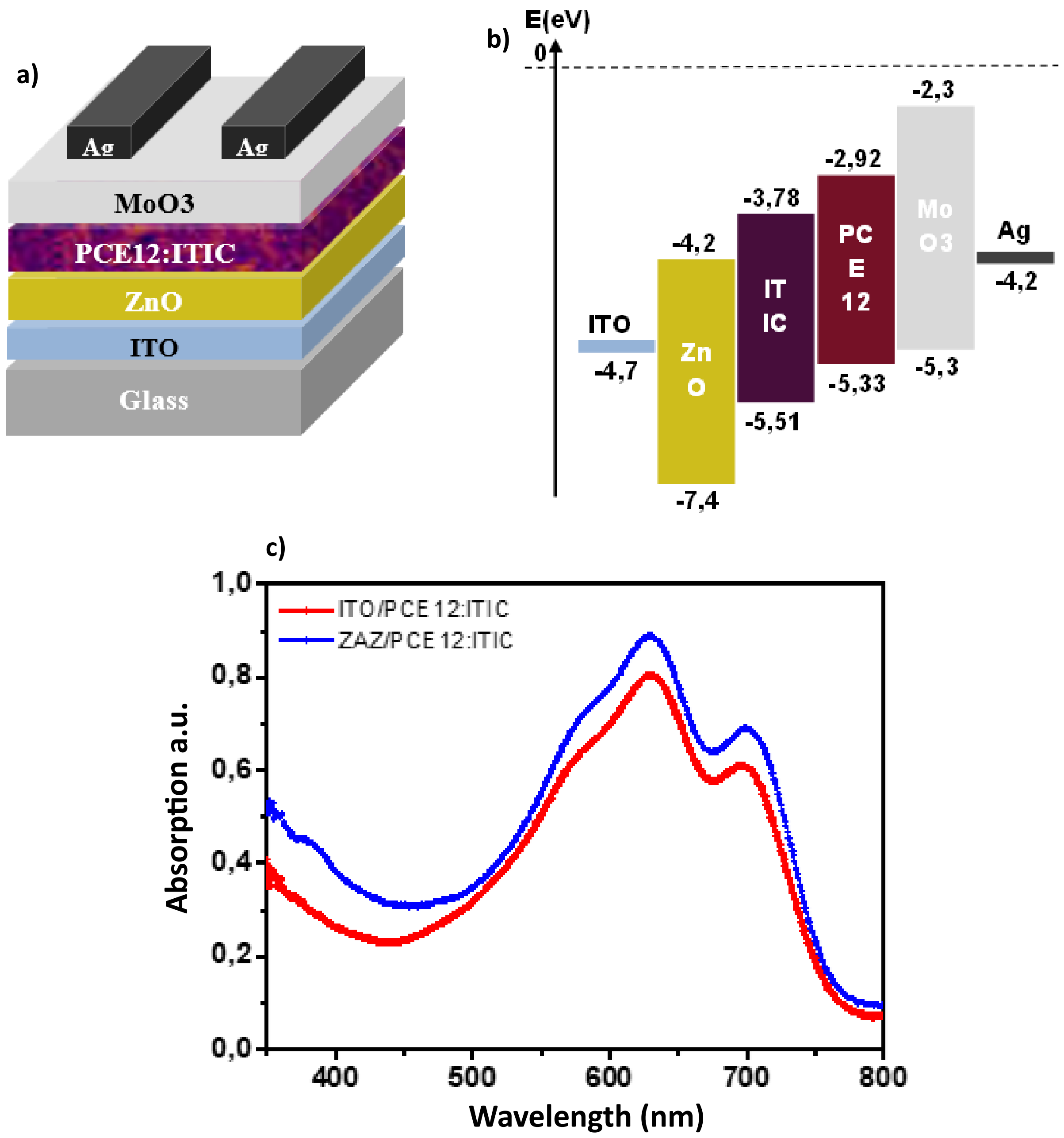

Figure S1: a. configuration of the solar cell with the different interface layers, b. energy level of the materials used in the device [1], c. absorption curves for the active layer (210 nm) on ZAZ and ITO.

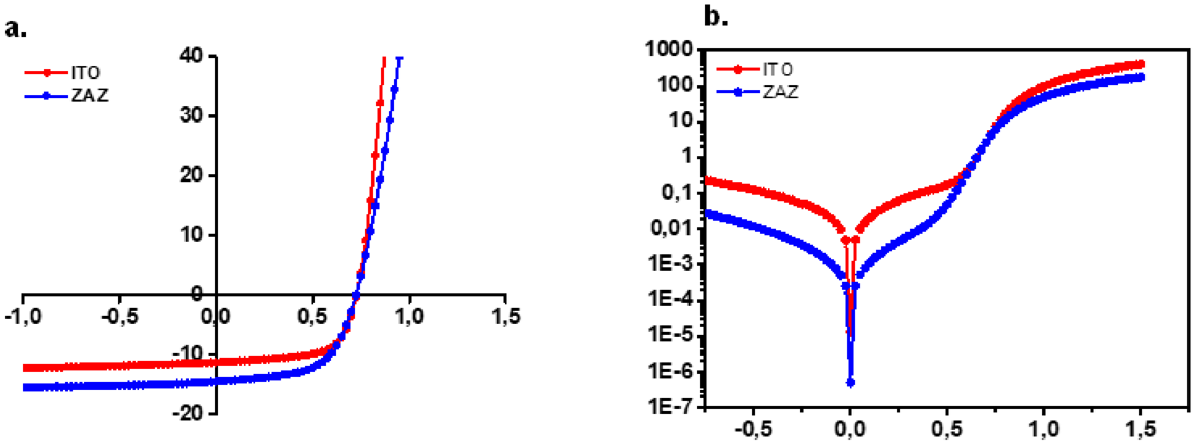

Figure S2: J(V) curves for PF2:PC71BM(500nm) solar cells a. under solar simulator (100mW.cm-2) ; b. in dark conditions.

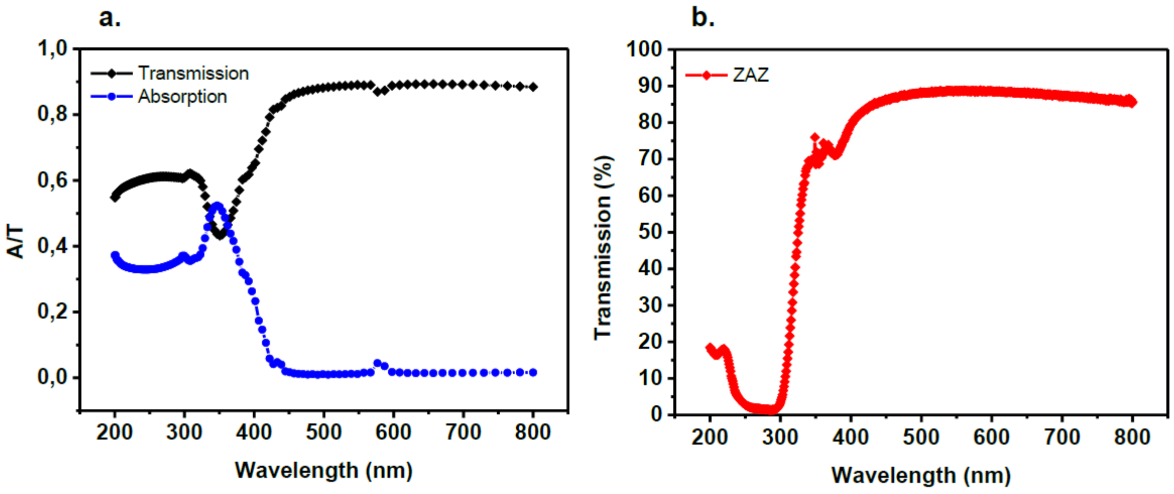

Figure S3: a. Absorption and transmission curves of a ZAZ electrode modeled with 5 AgNWs; b. transmission curve of an experimental ZAZ electrode.

References

1. W. Zhao, D. Qian, S. Zhang, S. Li, O. Inganäs, F. Gao, and J. Hou, Fullerene-Free Polymer Solar Cells with over 11% Efficiency and Excellent Thermal Stability, *Adv. Mater.*, vol. 28, nº 23, p. 4734-4739, **2016**, doi:0.1002/adma.201600281.
